# Melatonin promotes cytotoxicity while reducing cell motility and antioxidant defenses in ovarian cancer cell lines

**DOI:** 10.1016/j.toxrep.2025.102149

**Published:** 2025-10-28

**Authors:** Henrique Spaulonci Silveira, Roberta Carvalho Cesário, Vinicius Augusto Simão, Fernando Guimarães, Fábio Rodrigues Ferreira Seiva, Debora Aparecida P.C. Zuccari, Glaura Scantamburlo Alves Fernandes, Milena Cremer de Souza, Russel J. Reiter, Luiz Gustavo de Almeida Chuffa

**Affiliations:** a Department of Structural and Functional Biology, UNESP - São Paulo State University, Institute of Bioscences, Botucatu, São Paulo 18618-689, Brazil; b Hospital da Mulher “Professor Doutor José Aristodemo Pinotti” – CAISM, UNICAMP, Campinas, São Paulo, Brazil; c Department of Chemistry and Biochemistry, UNESP - São Paulo State University, Institute of Bioscences, Botucatu, São Paulo 18618-689, Brazil; d Faculdade de Medicina de São José do Rio Preto, São José do Rio Preto, São Paulo 15090–000, Brazil; e General Biology Department, Biological Sciences Center, State University of Londrina, Londrina, Paraná, Brazil; f Department of Cellular and Structural Biology, UTHealth, San Antonio, TX 78229, United States

**Keywords:** Ovarian cancer, Melatonin, Oxidative stress, Antioxidant defense, Catalase, Cell invasion and migration

## Abstract

Ovarian cancer (OC), a highly recurrent and fatal tumor, poses diagnostic challenges due to generic symptoms and chemoresistance. Melatonin (Mel) is an indoleamine acting against tumor progression and exhibiting pro-oxidative actions in tumor cells. This in vitro study explores the impact of Mel on antioxidant defenses of OC cells (high-grade SKOV-3 and low-grade CAISMOV-24 lines), focusing on its receptor-dependent and -independent effects. Cell viability was evaluated through MTT assay and antioxidant system was assessed in supernatants by measuring glutathione (GS), reduced (GSH) and oxidized (GSSG) glutathione, catalase (CAT), glutathione S-transferase (GST), and superoxide dismutase (SOD). Mel accumulated intracellularly and exerted cytotoxic effects, reducing cell viability in both cell lines. Notably, Mel independently of its membrane receptors, inhibited migration and invasion, thus showing its anti-tumoral potential. By investigating melatonin’s actions, we observed an impact on the antioxidant system primarily through the reduced activity of CAT and the GS axis. The modulation of these antioxidants by Mel demonstrates its multifaceted role in OC, emphasizing its therapeutic potential. We also demonstrated, for the first time, the theoretical ability of Mel to bind to CAT, which may be responsible for the reduction in enzyme activity. This study provides novel insights into Mel's receptor-independent actions and supports its potential as an adjuvant therapeutic agent in OC.

## Highlights

● Melatonin reduces viability and progression in high- and low-grade OC cells.
● Melatonin modulates antioxidant defenses, targeting catalase and gluthathione axis.
● Melatonin acts via receptor-independent pathways in OC cells.
● Melatonin binds near catalase’s heme group, influencing its activity.

## Graphical Abstract

**Figure:**
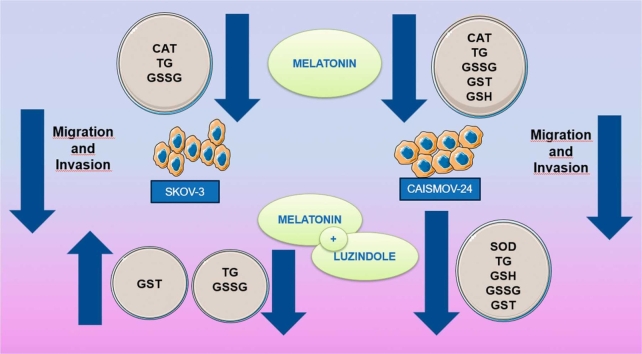

## 1 Introduction

Ovarian cancer (OC) is a common female tumor subtype characterized by high recurrence and mortality, largely due to late diagnosis and aggressive recurrence despite treatment and surgical removal [1], [2], [3]. Modifications in cellular metabolism occur when tumors redefine how they obtain nutrients and navigate metabolic routes to fulfill the requirements for biological energy, duplication, and the control of reactive oxygen species (ROS) levels in the cancer cells [4]. These changes are recognized as key aspects of cancer metabolism, marked by features such as tissue invasion and metastasis, loss of growth inhibition, rapid proliferation, sustained cell viability, resistance against cell death, initiation of the angiogenesis pathway, evasion of the immune response, and modified cellular vitality [5], [6]. Although OC is characterized by high recurrence and mortality rates, current therapies remain limited by chemoresistance and the lack of effective adjuvant agents. These unmet clinical needs highlight the potential relevance of exploring melatonin as a complementary therapeutic option.

Melatonin (Mel; N-acetyl-5-methoxytryptamine) is a lipophilic molecule converted from serotonin by the enzymes arylalkylamine N-acetyltransferase (AANAT) and acetylserotonin O-methyltransferase (ASMT) [7]. Mel has been characterized by actions that may either depend on or be independent of its membrane receptors (MT1 and MT2). Notably, receptor-independent effects of Mel have been demonstrated in different cancer types [8], [9]. While its primary synthesis occurs in the pineal gland during periods of darkness, other reports have shown its productions in different tissues and cells with local actions [10]. Mel has numerous functions under normal metabolic conditions including its antioxidant actions by removing reactive oxygen species (ROS) and reactive nitrogen species (RNS) and stimulating DNA repair mechanisms [11]. In many cancer cell types, Mel has oncostatic functions associated with pro-oxidative function while concurrently reducing the migration and invasiveness of cancer cells and inducing apoptosis [12], [13].

Reactive species, including ROS and RNS, are normally generated during cellular metabolism and act as signaling molecules while influencing gene expression, cell growth, and transcription factor activation [14], [15], [16]. In healthy cells, there is a delicate balance between the production and removal of ROS, which ensures redox homeostasis and their optimal physiological effects [17].

In OC cells, elevated ROS from mitochondrial metabolism and NADPH oxidase activity contributes to tumor progression [18], chemoresistance and accelerated OC growth [19], and dysregulation of specific microRNAs (miRNAs) associated with OC malignancies [20], [21].

To counteract ROS-induced damage, cells rely on enzymatic antioxidants such as superoxide dismutase (SOD), catalase (CAT), and glutathione-related enzymes. The efficiency of these defenses depends on their localization and availability near the site of radical generation [22]. Mel is an antioxidant under normal circumstances but can serve a pro-oxidant under specific pathologies (Zhang et al., 2014). Although additional investigation is needed, Mel has been documented to promote an increase in ROS via different mechanisms [8], [23]. In head and neck cancer cells, Mel induced ROS by reversing mitochondrial electron transport chain (ETC) which generated the anti-growth behavior [24]. Mel also induces apoptosis mediated by the stimulation of ROS synthesis in breast cancer as well as in mesangial cells, thus documenting that the anti-cancer actions of melatonin may be mediated by excessive ROS generation [25], [23], [26]. To date, the role of Mel on oxidative processes in OC have not been examined. Therefore, the present study aimed to investigate the impact of Mel, via receptor-dependent and receptor-independent mechanisms, on the antioxidant defenses, migration and invasiveness of OC cells.

## 2 Materials and methods

### 2.1 Cell lines, culture medium, and reagents

The SKOV-3 cell line (ATCC® HTB-77) was purchased from the American Type Culture Collection (ATCC, Manassas, VA, USA), while the CAISMOV-24 cell line was kindly provided by the Women’s Hospital Prof. Dr. José Aristodemo Pinotti — CAISM (UNICAMP, Campinas, SP, Brazil). SKOV-3 cells were cultured in RPMI medium (Gibco, Paisley, UK), whereas CAISMOV-24 cells were maintained in DMEN/ F-12 (LGC, Cotia, Brazil). Both cell lines were supplemented with 10 % fetal bovine serum (FBS) and antibiotics, including penicillin (100 IU/mL) and streptomycin (100 µg/mL) (Gibco). Cultures were incubated in a humidified chamber at 37°C with 5 % CO_2_. Cellular expansion was carried out in 75 cm^2^ and 25 cm^2^ culture flasks (Costar, Cambridge, MA, USA). Upon achieving 80 % of confluence, the culture medium was carefully removed, and the cells were washed twice with 10 % phosphate-buffered saline (PBS; Oxoid Limited, Hampshire, UK). Subsequently, trypsin/EDTA (Gibco) was employed to interrupt adhesion to the flasks.

### 2.2 Experimental schedule and melatonin treatment

To investigate the impact of Mel and its combination with luzindole (Luz) on OC cells, we initially determined the safe concentrations using the IC_50_ method (MTT assay). The IC₅₀ of Mel was calculated by exposing cells to increasing concentrations, normalizing absorbance to controls, and plotting cytotoxicity verus concentration; a linear regression of the dose-response curve identified the Mel concentration causing 50 % cell death. Based on these results, Mel was administered at 3.4 µM for SKOV-3 cells and 7 µM for CAISMOV-24 cells. For both cell lines, the concentration of Luz, a Mel receptor antagonist, was set at 1 µM. To assess the impact of Mel in combination with Luz, each OC cell line was divided into three different experimental groups: Control: cells cultured in a medium containing 100 µL of DMSO solution (1 % DMSO+EtOH as vehicle) without treatment; Mel: cells treated with Mel at concentrations varying from 2 to 7 µM (SKOV-3 and CAISMOV-24), plus vehicle; and Mel + Luz: cells treated with a combination of Mel and Luz, plus vehicle. In the combination group, Luz was administered first, followed by pre-determined Mel concentration after 30 min. In all assays, cells were exposed to Mel and/or Luz for a total duration of 24 h. Both compounds were dissolved while maintaining the molarity values specified by the manufacturers. All experiments were conducted in triplicate at both the biological and technical levels.

### 2.3 Cell viability assessment

Cell viability was evaluated using the MTT assay to determine the appropriate Mel concentrations based on the IC_50_ values. Once reaching the desired confluence, SKOV-3 and CAISMOV-24 cells were detached using trypsin, seeded into 6-well plates at a density of 5 × 105 cells per well, and cultured in the appropriated medium supplemented with 10 % FBS. Once the cells adhered, Mel and Luz were added to culture medium according to the experimental schedule, at defined concentrations of 2.0, 3.4, 5.0, and 7.0 µM. Next, the viability curves were generated based on MTT solution (5 mg/mL) following 24 h of treatment. To assess cytotoxicity, the assay was replicated in 96-wells plate, and absorbance was measured using a microplate reader (Epoch, Bio Tek Instruments, USA).

### 2.4 Cell invasion and migration assays

The evaluation of SKOV-3 and CAISMOV-24 cell invasiveness was performed using 24-well plates. A thin membrane of Geltrex® was added to each well, occluding the lower polyethylene terephthalate (PET) membrane. The OC cells (1 ×10^5^) were added to the top of the insert and received standard medium without FBS. The invasive potential was analyzed based on the ability of cells to cross the gel barrier and the PET membrane through the pores (8.0 µm), being attracted chemotactically by inferior coverage of culture medium containing 5 % FBS. The plates were placed in a CO_2_ atmosphere at 37ºC for 24 h. After incubation, SKOV-3 and CAISMOV-24 cells were fixed in methanol for 10 min, and the remaining cells were removed by scraping. Migrated cells were stained with a 0.1 % toluidine blue solution and photographed with a 5X objective in an inverted microscope (ZeissAxiovert®). For migration assay, a similar experimental procedure was used, except for Geltrex® which was not added to the transwell chamber. All experiments were assayed in triplicate based on four fields and submitted to automatic cell count LUNA-II® (Logos Biosystems, South Korea)

### 2.5 Measurement of melatonin concentration

Following treatment, SKOV-3 and CAISMOV-24 cells were lysed, and Mel levels were quantified using a pre-coated human-specific commercial ELISA kit (EH3344, Fine Test) following the manufacturer’s instructions. Absorbance was measured at 450 nm using a microplate reader (Epoch, BioTek Instruments, USA). Mel concentrations were determined by interpolating the absorbance values from standard curves generated by plotting standard concentrations against their respective absorbance readings. The assay range varied from 7.813 to 500 pg/mL, with a sensitivity threshold of 4.688 pg/mL. Intra- and inter-assay precision of the kit was assessed using low, medium, and high concentration samples. Intra-assay CVs ranged from 4.84 % to 5.25 %, and inter-assay CVs ranged from 5.1 % to 5.16 %, demonstrating good assay reliability. Mel concentrations were expressed in pg/mL.

### 2.6 Preparation of the supernatant for analyzing the antioxidant system

After Mel treatment, the culture medium was discarded, and cells were washed with PBS. Cell lysis was performed through three freeze-thaw cycles, alternating between −80 ºC and 37 ºC for 30 min each. Protein levels were then quantified and used to normalize the results for total glutathione (GS), reduced glutathione (GSH), oxidized glutathione (GSSG), catalase (CAT), glutathione S-transferase (GST), and superoxide dismutase (SOD).

### 2.7 Activity of SOD and CAT

After treatment with Mel and Luz, the cells (5 ×105 cells/mL) were washed with PBS (pH 7.4) and re-plated for all the analyzes. SOD enzyme activity assay was performed according to Marklund and Marklund [27] with some modifications. The reaction medium consisted of methionine (13 mM), riboflavin (4 mM), and NBT (75 µM) in phosphate buffer (50 mM, pH 7.8). A 10 µL aliquot of the cell extract was added, and the samples were subjected to fluorescent light (13 W) for 5 min. Control samples (lacking enzymes) and a blank (containing all reagents but not exposed to light) were included. Absorbance was measured at 560 nm and 25°C using a MultiSkan-Sky|High microplate reader. For CAT activity assessment, cells were lysed, and the reaction medium was prepared according to [28]. Absorbance was measured at 240 nm using a spectrophotometer for 1 min, with readings taken at 15-second intervals. The absorbance values were normalized to the control group.

### 2.8 Measurement of GT, GST, GSH, and GSSG

The levels of reduced glutathione (GSH) and total glutathione (GT) were quantified following a modified version of the protocol proposed by Rahman et al. [29], The assay involved the reaction of 5,5′-ditiobis 20-nitrobenzoic acid (DTNB) with the homogenate, resulting in a yellow-colored product. Total glutathione (TG) levels were determined using DTNB, nicotinamide-adenine dinucleotide phosphate (NADPH), and glutathione reductase in the homogenate. Absorbance for GT and GSH was measured at 412 nm using a Multiskan GO microplate reader (Thermo Scientific), and data were expressed as µmol/mg protein. GSSG levels were calculated based on reaction stoichiometry [30]. Glutathione S-transferase (GST) activity was assessed using 1-chloro-2,4-dinitrobenzene (CDNB) in a potassium phosphate buffer. Absorbance was measured at 340 nm over 160 s, with five readings taken at 40-second intervals [31]. The absorbance values were normalized to the control group.

### 2.9 In silico molecular blind docking

The target protein used in this *in silico* study was human liver mitochondrial CAT (PDB ID: 8SGV), which was downloaded from the Research Collaboratory for Structural Bioinformatics Protein Data Bank (RCSB PDB) database (https://www.rcsb.org/; accessed on 10 January 2025) [32]. CAT is a tetramer composed of four identical subunits (A,B,C, and D) with over 500 amino acid residues. Each chain is coordinate with iron-heme ring and binds NADPH as a cofactor. We conducted our analysis considering the A subunit isolated. The preparation of the protein for the molecular docking was conducted in the UCSF ChimeraX software (version 1.8) [33]. Water molecules, nonstandard residues and the chains B, C and D were deleted. The 3D structure of melatonin molecule (CID: 896) was downloaded from PubChem (https://pubchem.ncbi.nlm.nih.gov/; accessed on 10 January 2025) [34] and was prepared in the Avogrado software (version 1.2.0) by adding hydrogens and optimizing the molecule geometry. The next steps were done in the Autodock Tools software (MGLTools-1.5.6). In that stage, any residual water molecule was deleted, polar hydrogens and Kollman charges were attached in the protein. The melatonin structure was forced to let all rotatable bonds rotatable. The grid box was set to 126, 126, and 126 Å along the X-, Y-, and Z-axis with a grid spacing of 0.925 Å in order to recognize the whole CAT subunit. The AutoDocking parameters adopted were: GA runs = 500; population size = 150; maximum number of energy evaluations = 25.000.000; GA crossover mode = two points. The Lamarckian Genetic algorithm was selected to search for the best conformations and the lowest binding energy conformer was chosen for further analysis. The Chimera X and the Biovia Discovery Studio Visualizer (version 24.1.0) were used to analyze and visualize the docking results.

### 2.10 Statistical analysis

To assess differences among groups, all data were analyzed, with normality tests performed prior to one-way analysis of variance (ANOVA). When statistically significant differences were detected, Tukey’s post hoc test was applied for multiple comparisons. Results are expressed as the mean ± standard deviation (SD), with statistical significance set at P < 0.05. Data analysis and graph generation were performed using GraphPad Prism 9.0 (GraphPad Software, San Diego, CA, USA), while MTT assay results were processed in R. All experiments were conducted in biological and technical triplicates to ensure reproducibility and reliability of the results.

## 3 Results

### 3.1 Mel treatment induces cytotoxicity and enhances its intracellular accumulation in OC cells

**Fig. 1:**
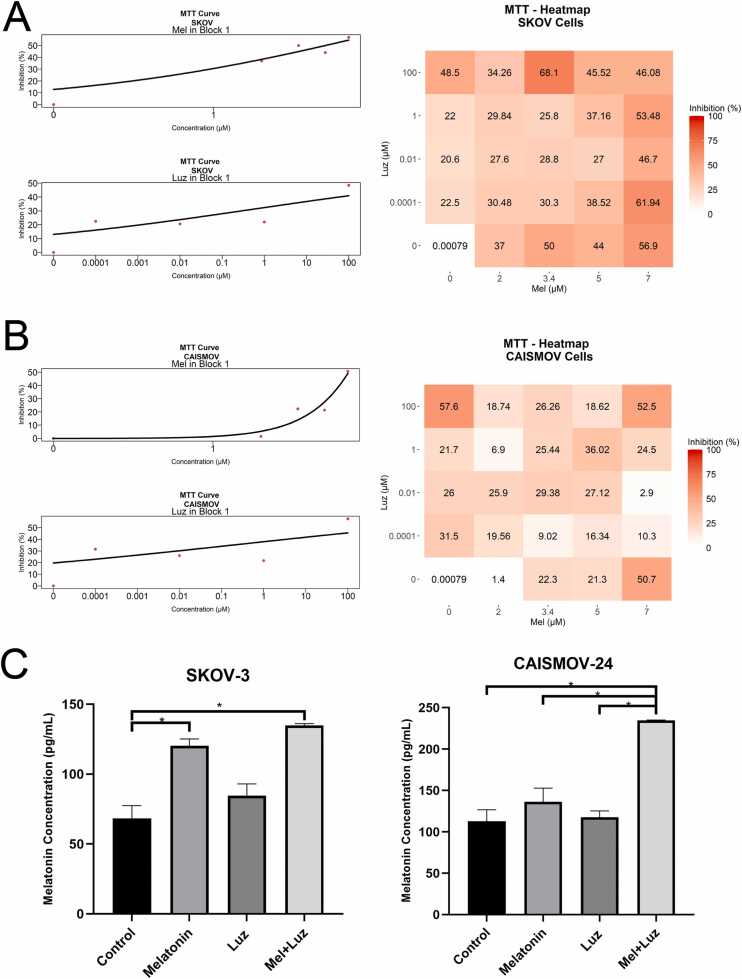
Cell viability and intracellular melatonin concentration following treatment with Mel and Luz in SKOV-3 and CAISMOV-24 cells. A) MTT assay results in SKOV-3 cells expressed as percentage of inhibition; curve analysis and synergy test. B) MTT assay results in CAISMOV-24 cells expressed as percentage of inhibition; curve analysis and synergy test. C) Intracellular melatonin levels measured in extracts of both cell lines. Data are presented as mean ± SD of triplets. * P < 0.05.

Based on MTT curve analysis, we confirmed the cytotoxic effect of Mel, with previously established IC_50_ values of 3.4 µM for SKOV-3 cells and 7 µM for CAISMOV-24 cells (Fig. 1A, B); these optimal concentrations were pre-determined in our earlier study using the same OC cell lines [35]. To evaluate whether Mel’s effects are mediated through its membrane receptors, we used luzindole, an MT1/2 receptor antagonist. After testing different concentrations, we selected 1 µM as the concentration with the lowest cellular toxicity in both cell lines. Based on the concentrations tested, the synergy analysis revealed that the combination of Mel and Luz resulted in lower cytotoxicity compared to Mel alone (Fig. 1A, B). The intracellular concentrations of Mel were measured in the OC cells by the enzyme assay. Mel treatment elevated the levels of intracellular Mel compared to the control group, especially in SKOV-3 cells. After blocking MT1/2 receptors, Mel promoted an increase in its own intracellular levels in both SKOV-3 and CAISMOV-24 cells (1.96-fold increase vs. Control and 2.08-fold increase vs. Control, respectively). More importantly, Mel alone in the presence of its receptors, increased its intracellular levels in SKOV-3 cells (1.75-fold increase vs. Control), thus restoring Mel concentration in OC cells where they are normally depressed (Fig. 1C). The increase in intracellular melatonin, especially after receptor blockade, suggests that its accumulation may act as a receptor-independent redox signal. Elevated melatonin could directly modulate oxidative balance influencing mitochondrial function and contributing to its anti-tumor effects in OC cells.

### 3.2 Mel reduces the migratory and invasive capacity of SKOV-3 and CAISMOV-24 cells regardless of the MT1/2 receptor activation

**Fig. 2:**
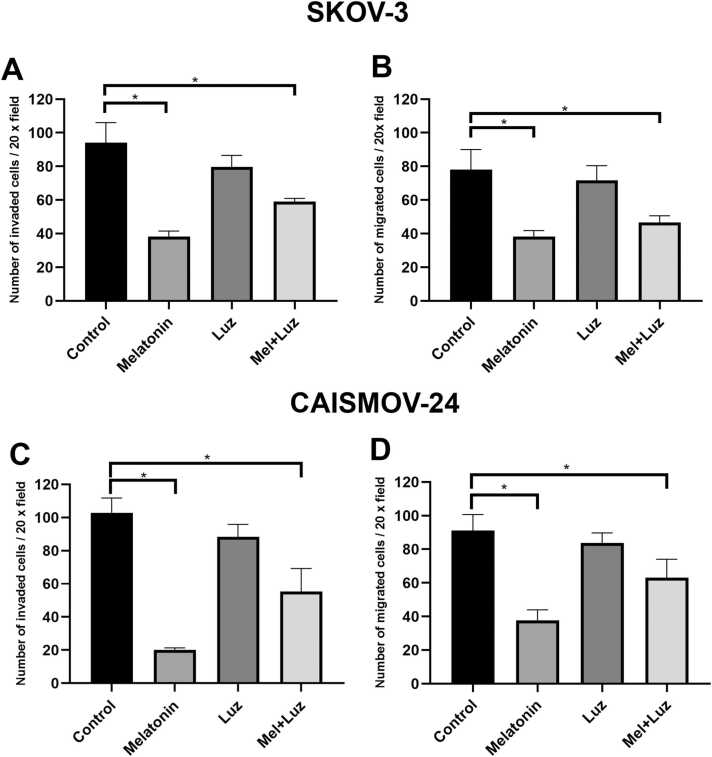
Effects of Mel and Luz on cellular invasion and migration. A) Invasive capacity of SKOV-3 cells after treatments with Mel and/or Luz. B) Migratory potential of SKOV-3 cells following the same treatments. C) Invasive capacity of CAISMOV-24 cells after treatments with Mel and/or Luz. D) Migratory potential of CAISMOV-24 cells following the same treatments. Mel: melatonin, Luz: luzindole. Data were expressed as mean ± SD of the triplets. *P < 0.05.

To investigate the effects of Mel and luzindole on the migratory and invasive potential of SKOV-3 and CAISMOV-24 cells, we used transwell inserts in 24-well plates. Mel alone significantly reduced the invasive potential of SKOV-3 and CAISMOV-24 cells by 2.45- and 3.58-fold compared to control groups, respectively. When combined with luzindole, the inhibitory effect was still evident, with a 2.17-fold decrease in SKOV-3 cells and a 2.38-fold decrease in CAISMOV-24 cells (Fig. 2A, C; Supplementary Figure 1A, B).

Similarly, Mel treatment alone significantly suppressed cell migration in both SKOV-3 and CAISMOV-24 cells, with reductions of 2.03- and 2.29-fold relative to controls, respectively (Fig. 2B, D; Supplementary Figure 2A, B). The combination of Mel and luzindole also led to a marked decrease in migration, with a 1.67-fold reduction in SKOV-3 cells and a 2.02-fold reduction in CAISMOV-24 cells compared to controls. These findings suggest that Mel effectively inhibits the migratory and invasive capacity of OC cells, even in the presence of MT1/2 receptor blockade.

### 3.3 Mel differentially regulates the antioxidant enzymes in OC cells

**Fig. 3:**
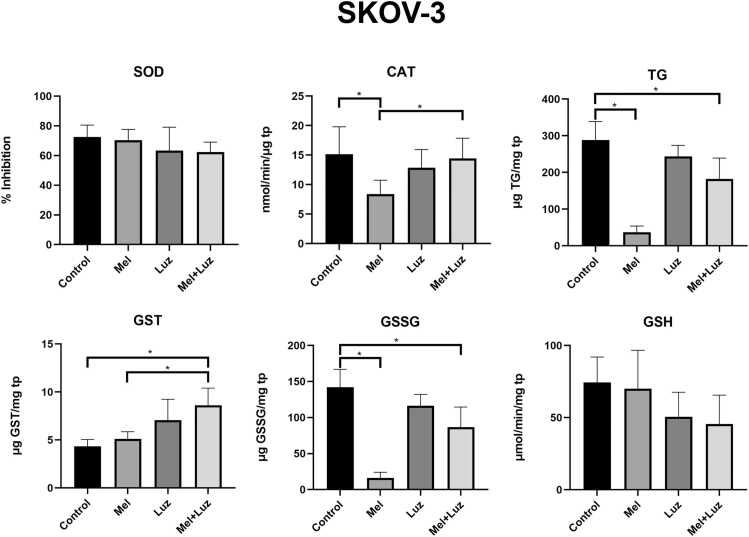
Enzymatic activities of SOD, CAT, and GST, and content of GT, GSSG, and GSH in SKOV-3 cells following 24 h of treatment with Mel and luzindole. Data are presented as mean ± SD of the triplets, with * p < 0.05. All samples were analyzed in triplicate within the same experimental run. Statistical analysis was performed using One-way ANOVA complemented by Tukey’s post hoc test. SOD: superoxide dismutase, TG: Total Glutathione, GST: Glutathione-S-transferase, GSSG: Oxidized glutathione, GSH: Reduced glutathione, tp: total protein.

**Fig. 4:**
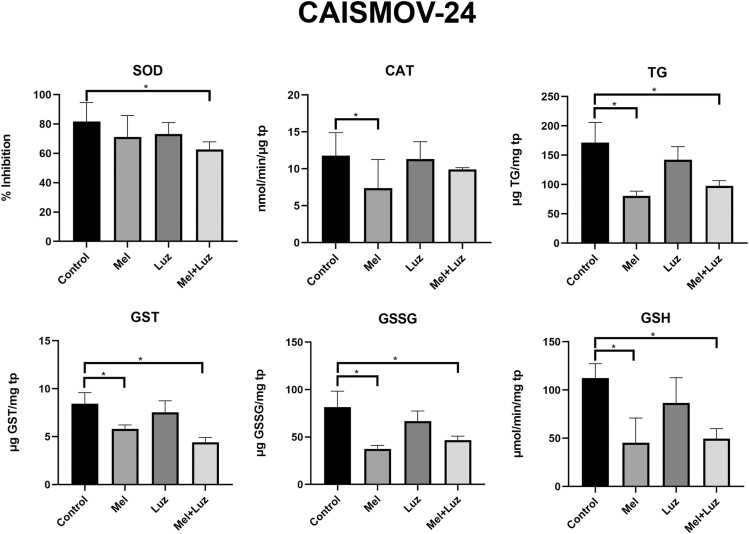
Enzymatic activities of SOD, CAT, and GST, and content of GT, GSSG, and GSH in CAISMOV-24 cells following 24 h of treatment with Mel and luzindole. Data are presented as mean ± SD of the triplets, with * p < 0.05. All samples were analyzed in triplicate within the same experimental run. Statistical analysis was performed using One-way ANOVA complemented by Tukey’s post hoc test. SOD: superoxide dismutase, TG: Total Glutathione, GST: Glutathione-S-transferase, GSSG: Oxidized glutathione, GSH: Reduced glutathione, tp: total protein.

SOD levels were significantly reduced only in CAISMOV-24 cells following treatment with Mel in combination with luzindole (p < 0.05). Regarding CAT levels, Mel treatment alone led to a significant reduction in both OC cells (p < 0.05 for CAISMOV-24 and SKOV-3). However, the presence of luzindole restored CAT levels to values close to those of the control groups, thus indicating a receptor-dependent response. This aligns with previous findings suggesting that melatonin’s effects on antioxidant enzymes are mediated through its receptors. The impact of Mel and luzindole on glutathione-related enzymes varied between the two OC cell lines (Fig. 3, Fig. 4). In SKOV-3 cells, Mel alone significantly decreased the levels of total glutathione (TG, p < 0.001) and oxidized glutathione (GSSG, p < 0.001) compared to the control group. The combination of Mel and luzindole also led to a significant reduction in TG (p < 0.01) and GSSG (p < 0.001). Conversely, this combination increased glutathione S-transferase (GST) levels compared to both the control (p < 0.001) and Mel alone (p < 0.01). Notably, CAISMOV-24 cells exhibited a different response. Mel alone or in combination with luzindole significantly reduced TG (p < 0.05), GST (p < 0.05), GSSG (p < 0.05), and reduced glutathione (GSH, p < 0.05) levels compared to the control group. These results suggest a differential redox regulation by Mel, with a pro-oxidative effect in CAISMOV-24 cells, highlighting its distinct impact on oxidative stress (OS) in OC cells.

### 3.4 Mel directly interacts with both the protein portion of CAT and its heme group

**Fig. 5:**
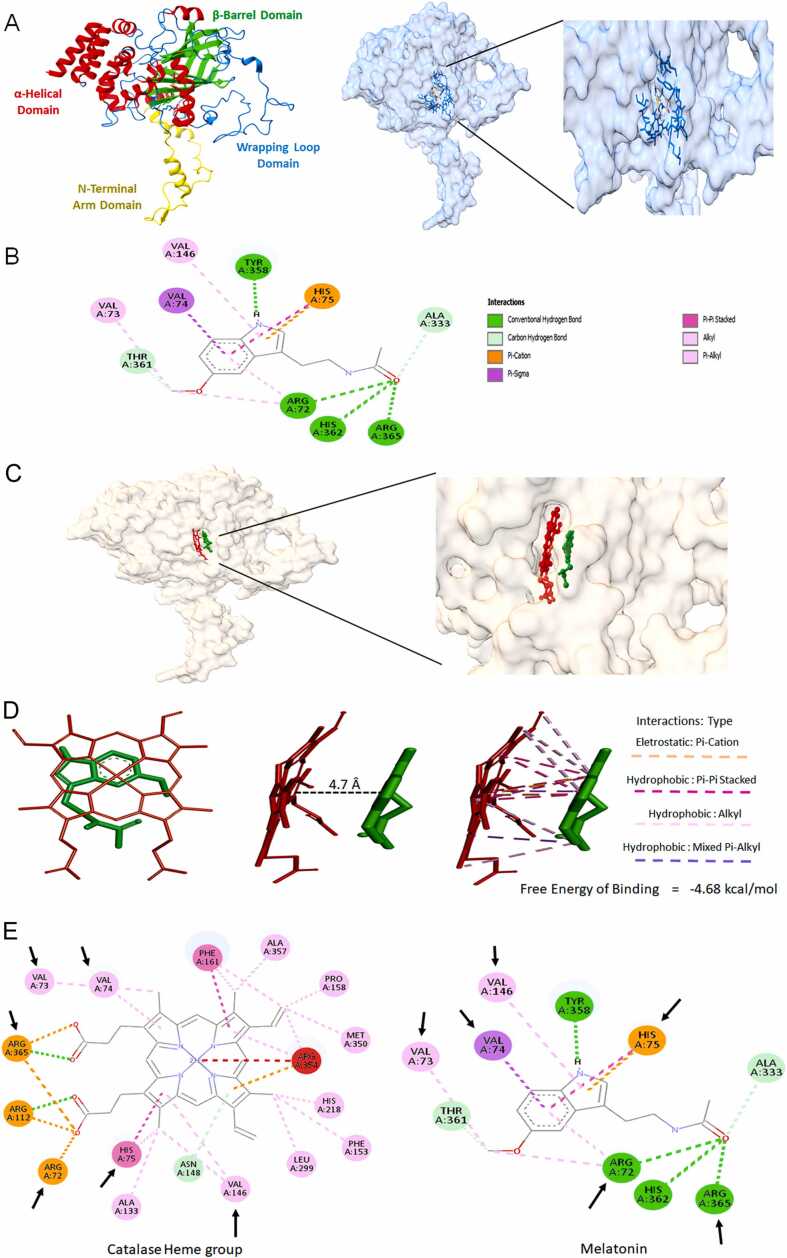
Molecular docking results showing the interaction between Mel and CAT. A) Structure of a CAT subunit evidencing its domains and Mel localization. B) Specific amino acids residues involved in Mel binding. C) Mel (green) binds in the same pocket as the heme group (red). D) Theoretical interaction between Mel and the heme group. E) Common amino acid residues (indicated by arrows) that form heme-binding site and may also contribute to Mel binding.

**Fig. 6:**
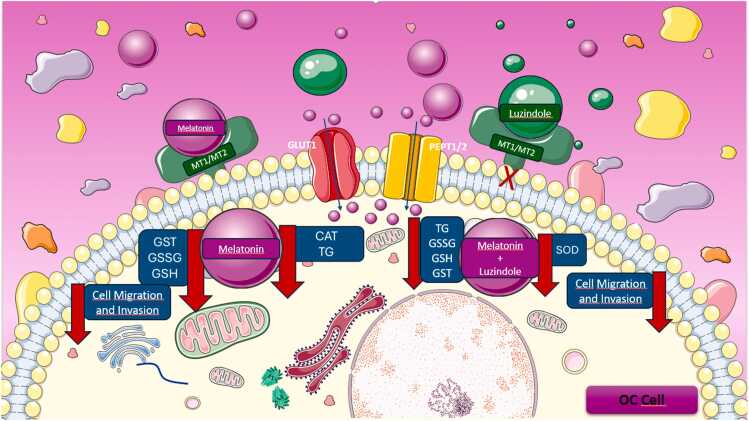
Schematic representation of the main findings underlying the effects of Mel alone or in combination with luzindole in OC cells. Mel modulates cellular redox homeostasis by attenuating several antioxidant enzymes while reducing the migratory and invasive potential of these malignant cells. When co-administered with luzindole (Luz), an MT1/MT2 receptor antagonist, Mel still weakened the cellular antioxidant machinery, retaining its anti-invasive effects, thereby indicating a predominant receptor-independent mechanism of action.

Given that Mel significantly reduced CAT levels in both OC cells, we next explored whether these effects could be related to a direct interaction between Mel and CAT. Through in vitro molecular docking analysis, the ability of Mel to interact with CAT was observed. Out of the 500 poses tested, the five most stable conformations exhibited binding energies ranging from –5.57 to –5.99 kcal·mol⁻¹ , with corresponding estimated inhibition constant (Ki) between 40.7 and 82.4 µM (Table 1). Subsequently, experiments were conducted to demonstrate the physical interaction between Mel and CAT. The most favorable interaction (Pose 453; –5.99 kcal·mol⁻¹) occurred within a pocket located in the β-barrel domain, near the heme-binding site (Fig. 5A). Mel directly interacts with the polypeptide chain of CAT through hydrogen bonds involving residues Arg72, Tyr358, His362, and Arg365. Additionally, alkyl interactions with Val73, Val74, and Val146, as well as π-interactions with His75, are also observed (Fig. 5B). As Mel was found to be positioned near the porphyrin ring (Fig. 5C), we further docked the interaction between these two molecules. The results show a close distance of approximately 7 Å between Mel and the heme group, suggesting that the structures are likely oriented in a specific way that facilitates interaction (Fig. 5D). Finally, it can be observed that some amino acids involved in anchoring the porphyrin ring to the protein may also interact with the indolamine (Fig. 5E). These potential interactions are of significant value and warrant further investigation. This structural evidence complements the biochemical findings, in which Mel decreased antioxidant enzyme activity and altered glutathione homeostasis, particularly showing a pro-oxidative profile in CAISMOV-24 cells. Together with its ability to reduce cell viability and suppress migration and invasion—even under MT1/2 receptor blockade—these results indicate that melatonin acts through both receptor-dependent and -independent pathways to modulate redox balance and impair the metastatic potential of OC cells (Fig. 6).

**Table 1:** Docking results for interaction between the Melatonin and Catalase (subunit A).

**Pose**	**BE**	**K**_**i**_	**vdW + Hbond + desolv**	**Electrostatic**	**Torsional**	**Unbound**
453	−5.99	40.74	−6.99	−0.19	+ 1.19	−0.49
82	−5.92	45.87	−6.66	−0.45	+ 1.19	−0.71
214	−5.72	64.28	−6.93	+ 0.02	+ 1.19	−0.38
146	−5.71	65.11	−6.93	+ 0.03	+ 1.19	−0.33
475	−5.57	82.37	−6.77	+ 0.01	+ 1.19	−0.37

BE: Binding energy; Ki: Estimated Inhibition Constant (micromole. L^−1^); vdW: van der Waals interactions; desolv: desolvation energy. Energy parameters are expressed as kcal.mol^−1^.

## 4 Discussion

We report that exogenously applied Mel increases its intracellular levels in OC cells, accompanied by reduced migratory and invasive capacities in a receptor-independent manner. Consistent with previous reports showing cellular uptake of Mel [36], [37], [38], the present findings suggest that Mel accumulation may occur via receptor-independent transporters such as GLUT1 and PEPT1/2. We also bring new information regarding the interference of Mel in the enzymatic antioxidant system of OC cells which may negatively impact their survival and growth. The increased intracellular level of Mel observed here may contribute to the antiangiogenic process, pro-oxidative, pro-apoptotic signaling, and metabolic reprogramming [39]. The stronger accumulation of intracellular Mel in SKOV-3 cells compared to CAISMOV-24 cells may reflect differences in cell type-specific factors, such as the expression of Mel transporters, receptor density, or endogenous synthesis machinery (e.g., AANAT, ASMT activities). By disinhibiting the pyruvate dehydrogenase complex (PDC) directly [40] or via downregulation of hypoxia-inducible factor 1-alpha (HIF-1α), Mel may restore oxidative phosphorylation, supporting mitochondrial Mel synthesis [37], [41].

Consistent with previous evidence [43], [42], our findings confirm that Mel reduces migration and invasion of OC cells, even when its receptors are pharmacologically blocked. Recently, our group showed that MT1 knockdown in SKOV-3 cells treated with Mel presented a remarkable reduction in cell migration and invasion [43]. Despite the use of concentrations ranging from 3.4 and 7 µM of Mel to SKOV-3 and CAISMOV-24 cells, respectively, our results clearly demonstrated a reduction in the invasive and migratory capacity of OC cells, regardless of the activation of Mel receptors. These differences suggest that Mel uptake and intracellular accumulation vary among cell types, distinct passages, depending on membrane composition, metabolic rate, and the efficiency of receptor-independent transport mechanisms. Although Mel reduced cell viability, the combination of Mel and luzindole showed lower cytotoxicity than Mel alone, yet migration and invasion were still significantly impaired. This indicates that the antimigratory and anti-invasive effects of Mel cannot be solely attributed to cytotoxicity. The involvement of MAPK and PI3K/AKT pathways has been reported as one of the major routes through which Mel exerts these anti-migratory effects in different cancer models, including ovarian, breast, and colon cancers [44], [45], [42]. These convergent findings suggest that Mel may suppress metastatic potential through broad interference with oncogenic signaling cascades.

Mitochondrial dysfunction can increase ROS and RNS, resulting in OS, which is countered by cellular antioxidant systems like SOD, CAT, and glutathione-related molecules [46]. An excessive and permanent generation of OS is related to genetic and epigenetic alterations that can promote tumorigenesis. However, paradoxically, increasing ROS and RNS levels in tumor cells has emerged as a strategy to induce their death [47], [48]. Mel contributes to this effect by modulating the cellular redox balance [47], [49]. While it acts as an antioxidant in healthy tissues, in tumor cells, Mel functions as a pro-oxidant, reducing their antioxidant defenses and thereby enhancing oxidative damage [50], [51].

Our findings further support the apparent pro-oxidant properties of Mel in OC cells, especially in CAISMOV-24 cells. We are the first to demonstrate a reduction in SOD activity when Mel was combined with luzindole, i.e., more sensitive to receptor-independent pathways in low-grade OC cells, and a significant reduction in CAT levels after Mel treatment in both OC cells. The observation that Mel alone reduced CAT levels in both cell lines, while Luz reversed this effect, supports a receptor-dependent mechanism. These findings reveal a cell line–specific modulation of redox homeostasis by Mel in OC cells. Mel treatments have also been associated with lower enzymatic activities of SOD and CAT accompanied by an increased level of ROS observed in human colorectal cancer cells and in the hepatocellular carcinoma cells [53], [52].

In healthy tissues, Mel generally upregulates antioxidant enzymes, including the glutathione system that plays a central role against OS [54], [49]. In both OC cells, Mel reduced total glutathione (TG) levels, though this effect was more pronounced in SKOV-3 cells. The reduced form of glutathione (GSH), essential for maintaining redox balance and often associated with chemoresistance [55], decreased in CAISMOV-24 cells treated with Mel, whereas SKOV-3 cells maintained GSH levels despite a significant reduction in GSSG. It is also reported that Mel can exert a depletion on GSH levels in HepG2 cells [47], [56], and in human myeloid leukemia cell line (U937) [57], [52]. This increased GSH:GSSG ratio suggests a more efficient antioxidant response in SKOV-3 cells, possibly reflecting higher resistance to OS.

Glutathione S-transferases (GST) and efflux pumps in tumor cells can diminish the efficacy of various chemotherapeutic agents, including cyclophosphamide [58], cisplatin [59], and others [60], by reducing their intracellular reactivity and promoting drug efflux. In this study, GST activity showed divergent responses. While in CAISMOV-24 cells both treatments decreased GST activities, in SKOV-3 cells the combination of Mel with luzindole increased its activity. Given that GST is regulated by the transcription factor Nrf2 [60], these results suggest that Mel’s effects on GST may depend on the oxidative environment and receptor status of the cell. Elevated GST and GSH levels have been linked to chemoresistance [61], while their depletion can sensitize tumor cells to oxidative damage. For instance, a recent study by [62] observed that the combination of Mel with cisplatin reduced cell viability, decreased GSH levels, and increased apoptosis in OC cells resistant to cisplatin, thereby revealing a promising strategy to overcome chemoresistance. In this context, treatment with Mel alone promoted a decrease in GST and GSH levels in CAISMOV-24, a well-established human low-grade serous OC cell line [63], but not in SKOV-3 cells, a non-serous OC cell line. In our previous study using the same OC cell lines and Mel concentrations, we observed a reduction in Warburg-type metabolism and potentially glutaminolysis, which further attenuated oncogenic targets associated with OC progression and invasion [35].

Mel is known to modulate antioxidant enzyme activity [64], [65], but the direct molecular interactions involved remain poorly defined. Given that CAT activity decreased in both OC cells, molecular docking was performed to explore Mel–CAT binding. Catalase’s catalytic mechanism occurs in two stages. Initially, the heme Fe^3+^ catalyzes the reduction of a hydrogen peroxide molecule to water, generating a covalent oxyferryl species and a porphyrin π-cation radical. This highly reactive radical subsequently oxidizes a second hydrogen peroxide molecule, converting it into molecular oxygen, while the ferryl oxygen species is released as water. Several amino acid residues, including tyrosine, play crucial roles in facilitating these reactions at the active site [66].

Molecular docking revealed that Mel binds within a structural pocket between β-sheet and wrapping domain, adjacent to the heme-binding cavity of CAT. The interaction is stabilized by hydrogen and hydrophobic bonds, with a binding energy of –5.99 kcal mol⁻¹ . Key residues involved include Arg72, Tyr358, His362, and Arg365, forming four conventional hydrogen bonds with Mel. The proximity of Mel to Tyr358, which interacts directly with the porphyrin iron, suggests a possible mechanism by which Mel interferes with CAT’s catalytic activity. These interactions may alter the microenvironment of the enzyme’s active site, contributing to the reduced CAT activity observed experimentally. These findings have potential therapeutic relevance: direct CAT inhibition by Mel may mimic or complement known CAT inhibitors that sensitize tumors to OS, supporting Mel’s role as adjuvant therapy. However, it is important to note that these interactions were predicted *in silico*, and it remains to be determined whether they occur at physiological concentrations of Mel. The binding of farnesiferol C, a sesquiterpene coumarin, to bovine liver CAT yielded similar findings, affecting both the enzyme’s affinity and catalytic activity [67]. The discovery of specific, non-toxic CAT inhibitors presents significant potential for use in cancer co-therapy. For instance, a Zn(II) complex tested against human colon cancer cells demonstrated cytotoxic effects by inhibiting CAT through mixed-type inhibition kinetics. Interestingly, the binding site of the complex also involved hydrogen bonding and exhibited a free energy of −7.13 kcal/mol [68], similar to the binding characteristics observed for Mel.

An unexpected result was the close spatial proximity of Mel to the heme group of CAT. Docking analysis showed a binding free energy of −4.68 kcal/mol between Mel and the porphyrin ring, indicating multiple interaction types. Several residues critical for maintaining heme stability – Val73, Val74, Arg72, His75, and Val146 – were involved in Mel binding. These interactions may contribute to melatonin’s inhibition of CAT activity, a possibility that warrants further investigation.

A key limitation of this study is the absence of siRNA- or shRNA-mediated silencing of Mel receptors, which would provide more conclusive evidence to differentiate receptor-dependent from receptor-independent mechanisms underlying the antioxidant effects of Mel. Additionally, the study did not directly assess intracellular levels of ROS or explore the potential pro-oxidant effects of Mel under varying cellular conditions. Another important point to consider is the lack of investigation into alternative Mel uptake pathways, such as GLUT1 and PEPT1/2 transporters, through the use of specific pharmacological inhibitors or gene silencing.

## 5 Conclusion

Collectively, Mel treatment attenuates the migratory and invasive capacity of OC cells while potentially enhancing its intracellular accumulation. Moreover, the antioxidant enzymatic defenses were dampened by Mel, particularly in CAISMOV-24 cells. Blocking MT1/2 receptors with the antagonist luzindole tended to mitigate these effects, suggesting partial receptor involvement. Based on our results, Mel predominantly exerts its effects on cell migration and invasion via receptor-independent mechanisms, although MT1/2 receptors may contribute partially in certain contexts. Importantly, Mel exhibits a dual role in OC cells, acting as a pro-oxidant in specific cell types. Additionally, these results provide new insights into Mel’s regulatory role in modulating redox balance in OC cells. Molecular docking results suggest potential interactions between Mel and CAT, which should be further validated using molecular dynamics simulations and enzyme kinetics assays to clarify the mechanisms of enzyme regulation. Overall, these data support the potential of Mel as an adjuvant therapy to reduce metastatic potential and modulate redox homeostasis in OC.

## Author contributions

Conceptualization, HS, FRS and LGAC; Data curation, HS; Formal analysis: RCC, FG, FRS, VAS, GSAF, MCS, DAPCZ and LGAC; Investigation, HS, FRS and DAPCZ; Methodology, HS, RCC, FG and DAPCZ; Visualization, RR; Writing – original draft, HS and LGAC; Writing – review & editing, RR. The authors read and approved the final version of this manuscript.

## CRediT authorship contribution statement

**Fernando Guimarães:** Methodology, Formal analysis. **Seiva Fábio:** Investigation, Formal analysis. **Simão Vinicius:** Formal analysis. **de Souza Milena:** Formal analysis. **Reiter Russel:** Writing – review & editing, Visualization. **Zuccari Debora:** Methodology, Investigation, Formal analysis. **Alves Fernandes Glaura:** Formal analysis. **Silveira Henrique:** Writing – original draft, Methodology, Investigation, Data curation, Conceptualization. **Cesário Roberta:** Methodology, Formal analysis. **Chuffa Luiz Gustavo:** Writing – original draft, Formal analysis, Conceptualization.

## Funding

This research was supported by CAPES (Coordenação de Aperfeiçoamento de Pessoal de Nível Superior – Brasil), grant number 88887.482443/2020-00, the National Council for Scientific and Technological Development (CNPQ, Process numbers 304108/2020-0 and 306117/2023-1 to LGAC), and the São Paulo Research Foundation (FAPESP grants #2021/12971-7 and #2025/03607-0 to LGAC).

## Declaration of Competing Interest

The authors declare the following financial interests/personal relationships which may be considered as potential competing interests: Luiz Gustavo de Almeida Chuffa reports financial support was provided by State of Sao Paulo Research Foundation. If there are other authors, they declare that they have no known competing financial interests or personal relationships that could have appeared to influence the work reported in this paper

## Data Availability

Data will be made available on request.
