# Supplementary material for: Melatonin promotes cytotoxicity while reducing cell motility and antioxidant defenses in ovarian cancer cell lines
Source: Toxicol Rep. 2025 Oct 28;15:102149. doi: 10.1016/j.toxrep.2025.102149 (PMC12621470; doi:10.1016/j.toxrep.2025.102149)
Supplement: Supplementary file 1 — Supplementary material [file mmc1.docx]

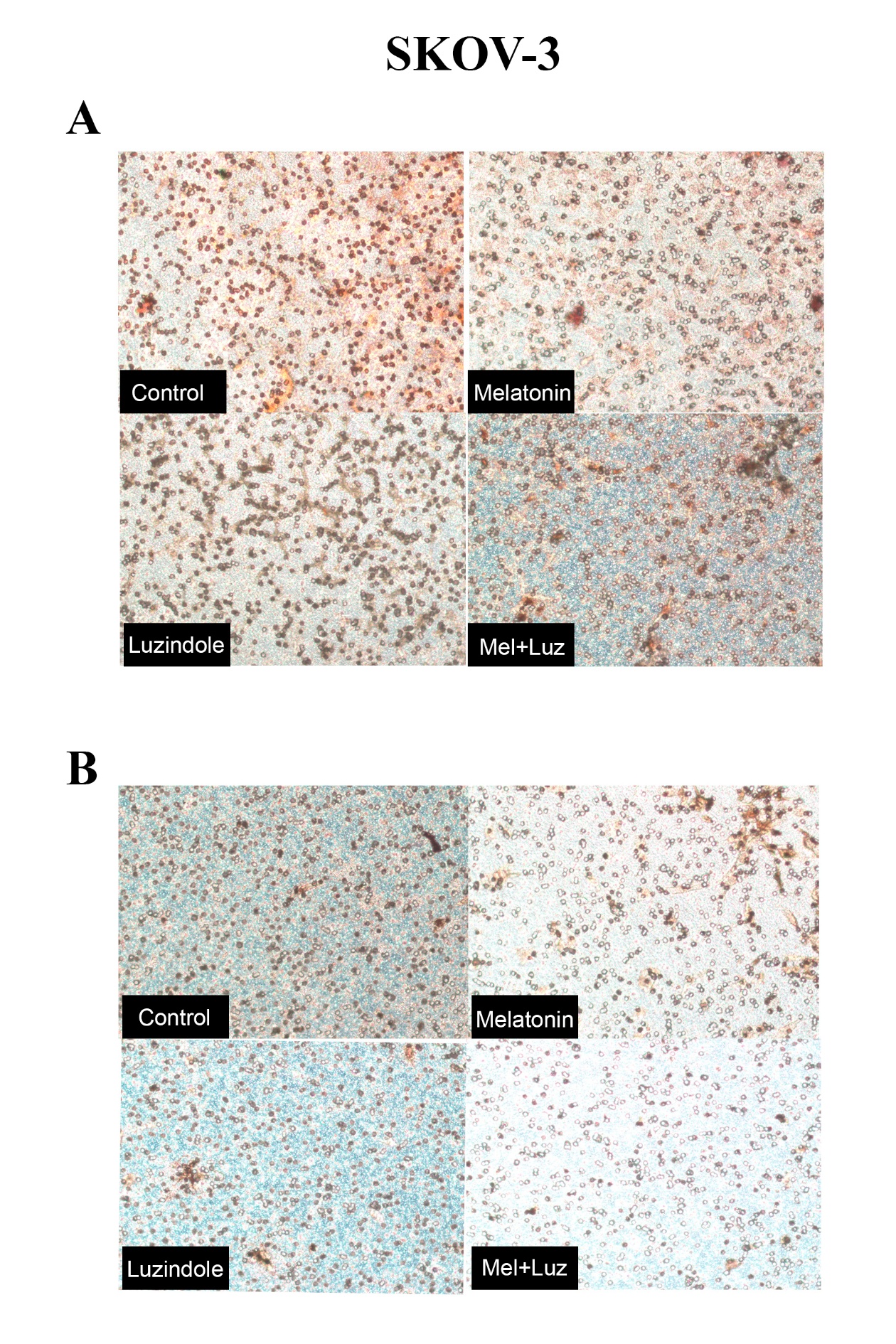


**Supplementary Figure 1.** Effects of Mel and Luz on SKOV-3 cells. A) Representative images showing SKOV-3 cells following treatment with Mel and/or Luz, illustrating their effects on invasive capacity. B) Representative images of cellular migration after treatments. Mel: melatonin; Luz: luzindole.


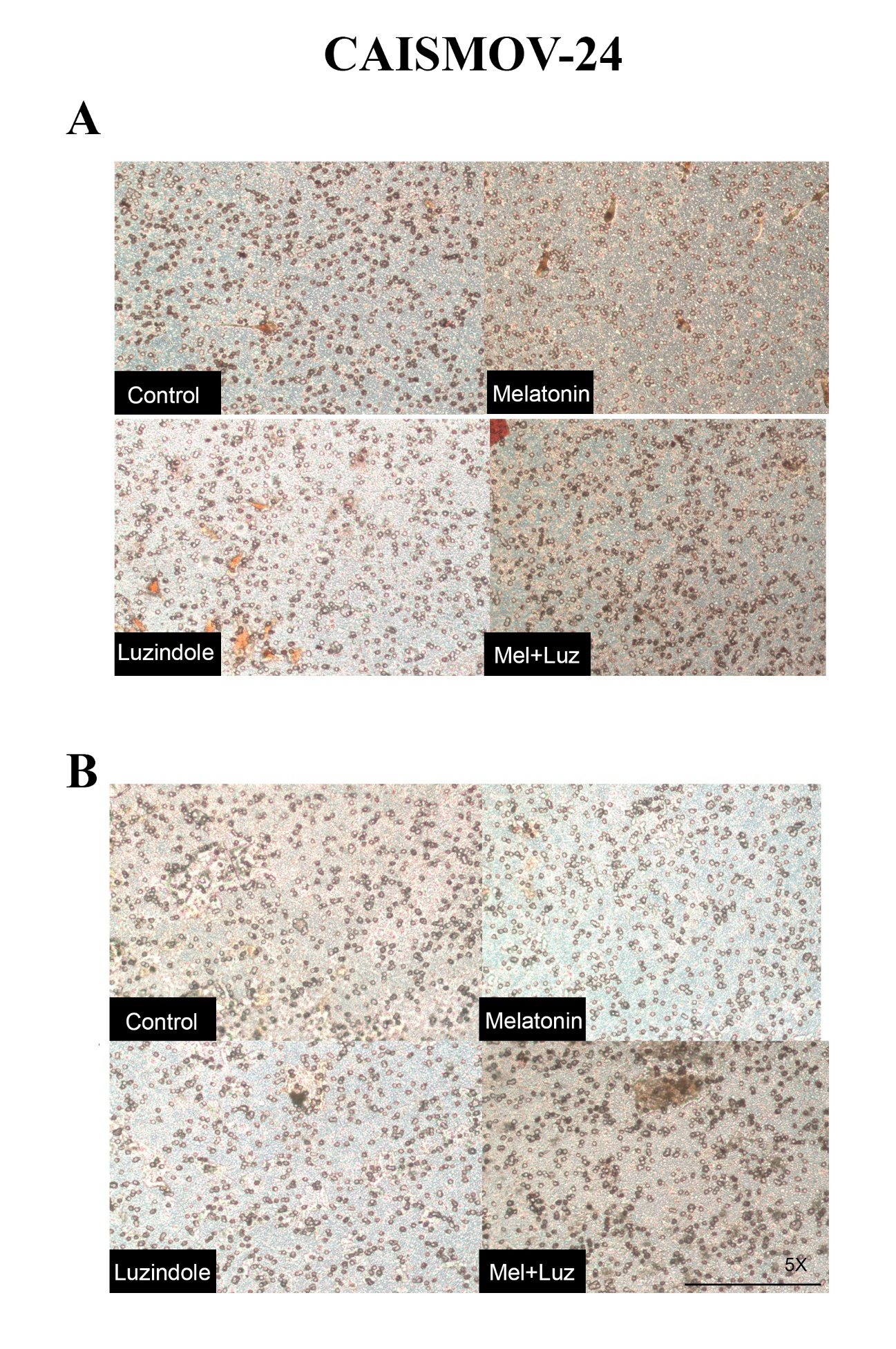


**Supplementary Figure 2.** Effects of Mel and Luz on CAISMOV-24 cells. A) Representative images showing CAISMOV-24 cells following treatment with Mel and/or Luz, illustrating their effects on invasive capacity. B) Representative images of cellular migration after treatments. Mel: melatonin; Luz: luzindole.
